# Knowledge, attitude, and utilization of traditional medicine within the plural medical system in West Java, Indonesia

**DOI:** 10.1186/s12906-024-04368-7

**Published:** 2024-01-30

**Authors:** Raden Maya Febriyanti, Kurniawan Saefullah, Raini Diah Susanti, Keri Lestari

**Affiliations:** 1 Department of Biological Pharmacy, Faculty of Pharmacy, Universitas Padjadjaran, Jalan Raya Bandung-Sumedang KM. 21, Jatinangor, Sumedang West Java, 45363 Indonesia; 2 Herbal Study Center, Universitas Padjadjaran, Jalan Raya Bandung-Sumedang KM. 21, Jatinangor, Sumedang West Java, 45363 Indonesia; 3 Faculty of Economy and Business, Universitas Padjadjaran, Jalan Raya Bandung-Sumedang KM. 21, Jatinangor, Sumedang West Java, 45363 Indonesia; 4 Faculty of Nursing, Universitas Padjadjaran, Jalan Raya Bandung-Sumedang KM. 21, Jatinangor, Sumedang West Java, 45363 Indonesia; 5 Department of Pharmacology and Clinical Pharmacy, Universitas Padjadjaran, Jalan Raya Bandung-Sumedang KM. 21, Jatinangor, Sumedang West Java, 45363 Indonesia

**Keywords:** Traditional medicine, Medical pluralism, Plural medical system

## Abstract

**Background:**

The concept of ‘medical pluralism’ has become more popular among scholars in applied health science and prevalent in societies where one medical system alone cannot adequately meet the health care needs of the entire population.

**Methods:**

The data collection is focused on the knowledge-belief-practice and the utilization of three medical systems in Kabupaten Bandung, West Java, Indonesia. Participants were purposively selected from households with at least one member experienced one of the listed diseases in the questionnaire. The extensive survey using a structured questionnaire has been undertaken to collect data on people’s health care utilization behaviour. The dataset is further analyzed using multivariate analysis through non-canonical correlation, with the analytical data provided by Statistical Package for Social Sciences (SPSS).

**Results:**

With regards to the total utilization by patients, the traditional medical system presents as the dominant medical system in the research area, accounting for 59.3% (*n* = 419) of total utilization, followed by the modern medical system (33.0%, *n* = 233), and transitional medical system (7.7%, *n* = 54). This study identified that village category, illness, illness duration, occupation, belief in traditional medicine, knowledge of modern medicine, accessibility, cost, proximity to the medical service, and insurance have significant (χ^2^ = 0.000) relationship with the utilization of medical systems. The results of the multivariate analysis show that the block of the predisposing socio-demographic factors and the block of the predisposing psycho-social factors correlate strongly with the utilization of medical systems.

**Conclusions:**

In general, people in Kabupaten Bandung, West Java, Indonesia seeks treatment from various sources, which in the context of the medical system, consists of the traditional, transitional, and modern medical system; therefore, it adopts the patterns of transcultural health care utilization. In terms of the knowledge, beliefs, and practices of traditional medicine in West Java, the inhabitants of the five research villages were commonly familiar with medicinal plants and speak profoundly about their knowledge of traditional medicine, which in the research area is perceived as accessible, efficacious, affordable and culturally appropriate with Sundanese community.

## Introduction

Indonesia is known as the country with the largest ethnic groups and cultures in the world and one of the world’s centers of biodiversity [1]. This rich potential of biological resources in Indonesia, integrated with knowledge of plant utilization by various ethnic groups in Indonesia, develops traditional knowledge systems including traditional medical knowledge or ethnomedicine [1, 2]. The utilization of traditional medicine in Indonesia is often in combination with biomedicine or modern medicine [3]. Furthermore, it is also noteworthy that over-the-counter (OTC) medicines – categorized as transitional medicine- also have remarkable persistence in Indonesia [3–5]. In a setting where several modern medical services are offered, the community also preserves their indigenous medical knowledge and practices, presenting variations in health-seeking behavior. Consequently, the use of traditional medicine, biomedicine, and OTC medicine by community members have established the current plural medical configuration, which is reflected in the co-existence of the traditional, transitional, and modern medical systems in Indonesia.

The idea of medical pluralism was developed in the context of research on countries where different kinds of medical systems exist alongside each other and interact with each other [6–8]. This phenomenon has been evident throughout the world and is particularly prevalent in societies where one medical system alone cannot adequately meet the health needs of the entire population [8]. The concept of ‘medical pluralism’ has become more popular among scholars in applied health science, not only because of the resurgence of traditional, complementary, and alternative medicine but also due to the crisis in public health care which requires governments to change their health care policies [9, 10].

Studies show that cultural belief is identified as an important factor for patients to choose specific medical services. Comprehensive studies for the assessment of risk factors, epidemiology, knowledge and attitude for kidney disease in Tanzania identify five major determinants in the use of traditional medicine, namely: health status, disease understanding, biomedical healthcare delivery, credibility of traditional practices, and strong cultural identities. Heterogeneous conceptions of disease across locales have been linked to unrealistic therapeutic expectations, perceived treatment failures, and subsequent nonadherence to prescribed medical regimens, thus increasing reliance on traditional medicines. Furthermore, individuals’ conceptions were found to be an amalgam of factors including the standard of biomedical healthcare, traditional health belief systems, the persistence of illness, and the manifestation of the disease via distinct symptoms. An individual’s general health status also significantly influence the choice to engage with traditional medicines. It is revealed that patients with diabetes, hypertension, cardiovascular diseases, and persistent edema are more inclined towards traditional medicines utilization. The decision to resort to traditional medicines is further influenced by the chronic nature and assumed severity of the medical condition. While the acceptability of traditional practices is deeply intertwined with strong cultural identities regarding traditional medicines, this acceptability is shaped by elements of cultural belief. Despite reservations concerning the scientific substantiation, quantification of doses, educational background of practitioners, and regulatory frameworks within traditional healing milieus, a substantial segment of the cohort retains profound cultural fidelity to traditional medicines [11, 12].

In the same fashion, Kasole et al. (2019) point out that the utilization of traditional medicine is influenced by cultural factors such as tradition, belief, and cosmovision and supporting factors such as economic considerations and ease of access. While the study mainly focus on the cultural factors in the utilization of traditional medicine for the treatment of diabetes mellitus as it is a complex health condition which also accompanied by various misconceptions and myths, the study also pointed out that the efficacy, availability, and accessibility of traditional medicines, alongside the economic considerations related to the cost of conventional medicines, and the influence of social networks, were identified as pivotal motivators for the utilization of traditional medicines. The study also reveals that a subset of participants engaged with traditional medicines as a cost-mitigation strategy against the expenses incurred from conventional medicines and as an alternative when economic limitations impeded access to conventional medical treatments [13].

In addition to the influence of cultural beliefs, the process of the choice of medical treatment is also influenced by the limitations of the environment which is conceptualized as accessibility [14–16]. In this context, Mulyanto et al. provide examples that medical decision-making may be associated with the cost of a particular treatment or the unavailability or inaccessibility of certain treatments. The study revealed supply-side elements, such as the accessibility and allocation of healthcare services, have been recognized as significant influences on the utilization of medical services. Moreover, recent analyses have highlighted significant variances in the healthcare infrastructure across the Indonesian regions. Consequently, people live in the cities engage to medical services more often than their conterparts [15].

As an ethnographic study of medical traditions, this study examines how the character of the Sundanese community is reflected in the healthcare-seeking process, particularly where several medical services co-exist within the community. Accordingly, by positioning the indigenous knowledge of traditional medicine and healing practices in relation to the utilization of the plural medical system, the present study seeks to provide an insight into the overall patterns of healthcare utilization in the Sunda region in West Java. This study presents the dynamics of medical pluralism and utilization of the medical system in Kabupaten Bandung, a rural district in West Java that is experiencing the impact of rapid urbanization from its surrounding districts.

## Methods

### Operationalization of the conceptual model

**Fig. 1:**
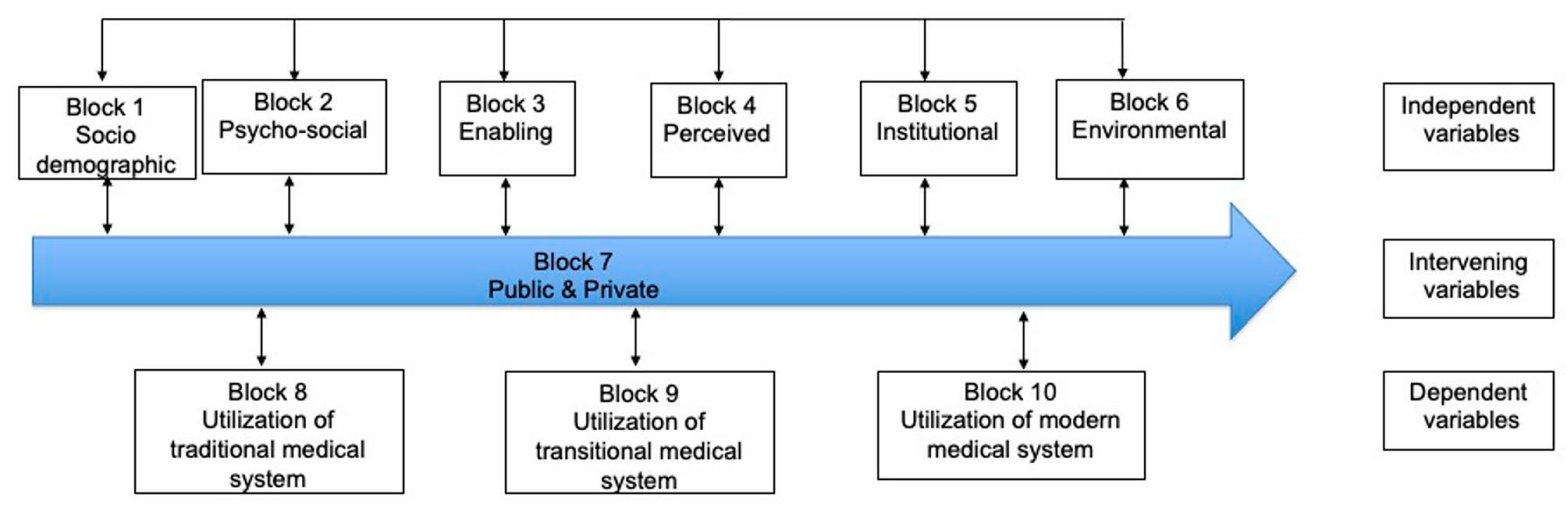
Conceptual model of transcultural health care utilization

The study adopts the ethnosystem approach developed and adapted by Slikkerveer (2006, 2019) within the context of medical pluralism in which the traditional, transitional, and modern medical systems co-exist within the community [17, 18]. As the basis for the analysis of patterns of health-seeking behaviour, blocks of independent variables, intervening variables, and dependent variables are conceptualized in the model of the mutual relation analytical model of transcultural health care utilization (Fig. 1).

Referring to the various studies conducted elsewhere in the field of medical anthropology and the health sector, and following the results of the pilot study, potentially significant variables on healthcare service utilization were identified. In the past, several studies have examined the role of predisposing factors, enabling factors, and need factors in the use of health services. A total of thirty-three (33) variables are identified for this study. Furthermore, the variables which have been identified are then formulated into measurable indicators. Indicators were identified based on the results of a pilot study in Kabupaten Bandung. After the indicators and answer categories were set, structured questionnaires were then developed. In the construction of the structured questionnaire, an indicator can ideally be measured with a single phenomenon. However, some indicators, i.e. socio-economic status and accessibility, are somewhat difficult to be measured with only one phenomenon. In this case, several questions were added to make sure the indicators were accurately measured [19].

#### Independent variables: predisposing factors

Predisposing factors describe the tendency of people to use health services, which can be predicted by individual characteristics which existed before the onset of the disease. The conceptual model in this study employs two types of predisposing factors: socio-demographic and psycho-social factors. Socio-demographic factors included in the present study are gender, age, level of education, place of birth, ethnicity, marital status, and occupation [20–22]. The second block in the predisposing factors is the psycho-social factors, which refers to the knowledge, ideas, opinions, beliefs and attitudes of the members of the community. This block includes the variables of knowledge, belief, and opinion which are directly linked with the three dependent variables.

Indicators of knowledge on medical systems cannot be measured with only single phenomena. Each variable was operationalized into several phenomena which are measured individually. In this case, respondents were asked to demonstrate their knowledge of the medical system by mentioning the name and use of medicinal plants, over-the-counter (OTC) medicine, and prescription medications including their application. The belief and opinion variables were measured by asking for the respondent’s perceived effectiveness and opinion on the medical system.

#### The enabling factors

The enabling factors relate to the ability of the people and the availability of the means to utilize health services. Enabling factors can be measured by family resources such as socio-economic status (SES), health insurance, family income, costs, free health care, and health insurance [21–23].

#### Perceived morbidity factors

Perceived morbidity was operationalized by the reported health status and diseases experienced by the respondent in the past twelve months. Ten diseases were listed in the questionnaire based on the highest prevalence in Indonesia from the categories of non-communicable and communicable diseases.

#### Institutional factors

The institutional factors comprised the financial and geographical accessibility of local and central health care facilities and services. This variable is measured with two phenomena related to the distance to access the facility of the particular medical system and the cost to obtain the medical service. Dimensions of distance to health care facilities, namely travel time, waiting time, and means of transport, could significantly influence health care utilization [14, 15, 24].

#### Environmental factors

Environmental factors describe the environmental characteristics where a respondent resides which may influence the utilization of the medical system. These factors are complicated in a large developing country. Access to health services is becoming increasingly unequal, particularly in rural areas [15].

#### Intervening factors

Intervening factors refer to the external factors which are considered as altering the standard patterns of behaviour. In the present study, the intervening factors are operationalised into several questions regarding perceived impact on the promotions of medical systems. Following the implementation of National Health care Coverage in 2014, public and private health insurance are also listed as intervening factors.

### Dependent variables

As the purpose of this study was to examine patterns and predictors of use *across* medical services (rather than strictly *within* one medical service), it is necessary to conceptualize these three health care systems as distinctive rather than joint components of one overall system, serving to reveal dimensions of medical pluralism and the extent to which medical dominance is (or is not) pivotal in this ‘new medical pluralism’. In the analysis, the dependent variables were divided into three utilization variables: the traditional, transitional, and modern medical systems. This categorization of medical system is defined as follows [17]:

- Traditional medical system based on the local perceptions, practices, and beliefs of a particular community;
- Transitional medical system characterized by the economic and financial interest of drug vendors and provided by the intermediary personnel in developing countries;
- Modern medical system as the official health care system which is based on Western science and technology and opposed to the traditional medical system.

### Selection of the research area

The current research was carried out in Kabupaten Bandung, located in the western region of Tatar Sunda on Java Island. This locale offers a rich bio-cultural landscape that serves as a pertinent backdrop for studying human-nature interactions. Alongside its geographical attributes, the site was selected taking into account environmental factors such as the dichotomy between rural and urban communities and the availability of local healthcare infrastructure. Utilizing a multistage cluster sampling technique, the study design incorporated stratification at each stage to optimize the sample. Specifically, sub-districts were categorized as either rural or urban. Lamajang, Sukaluyu, Cipanjalu, and Ciporeat were chosen as the rural representative villages, while Katapang, Soreang, and Baleendah represented the urban sectors.

### Selection of the samples

In this study, there are 620 households with at least one member who reported experienced one of the diseases in the questionnaire. Diagnosis of disease was based on self-reporting, and some participants provided the interviewer with health records from the clinics.

### Data analysis

Datasets were created for the analysis of the results of the household survey. After the dataset was prepared including some steps such as recoding and excluding the missing values, the next step taken was to perform the statistical analysis. In order to explain the relationship between two categorical variables, bivariate analyses with the cross-tabulation technique are applied to the defined independent and intervening variables, pairing them with the dependent variables. Pearson’s chi-square (χ^2^), Cramer’s tests are applied to each cross-tabulation [25]. All statistical analyses were performed using SPSS® Statistics for Windows™ version 26 by IBM® Corporation, Armonk, NY, USA.

## Results

### Characteristics of the respondents

The socio-demographic characteristics of the respondents in the research area constitute predisposing factors in the analytical model. The sample in this research consists of 706 respondents living among 620 households. Descriptions of the demographic characteristics of the samples are provided in Table 1.

**Table 1:** Subject characteristics (*n* = 706)

Characteristics		Rural area	Urban area	Total
		*N* = 384	*N* = 322	*N* = 706
Gender	Male	45.1%	41.6%	43.5%
	Female	54.9%	58.4%	56.5%
Age	0–17 years old	14.1%	2.5%	8.8%
	18–65 years old	40.4%	24.2%	33.0%
	66–79 years old	34.6%	54.7%	43.8%
	> 80	10.9%	18.6%	14.4%
Residential status	Native	83.3%	59.0%	72.2%
	Migrant	14.1%	38.2%	25.1%
	Temporary	2.6%	2.8%	2.7%
Education	No formal education	54.7%	30.1%	43.5%
	Basic education	29.2%	46.9%	37.3%
	Intermediate education	8.6%	18.3%	13.0%
	Higher education	7.6%	4.7%	6.2%
Marital status	Single	16.1%	5.9%	11.5%
	Married	72.4%	79.5%	75.6%
	Divorced/Widowed	11.5%	14.6%	12.9%
Occupation	Farmer	45.8%	0.0%	24.9%
	Construction worker	4.2%	5.0%	4.5%
	Retailer	12.8%	8.1%	10.6%
	Private Employee	2.9%	11.8%	6.9%
	Civil servant	1.8%	11.8%	6.4%
	Unemployed	32.6%	53.7%	42.2%
	Other	0.0%	9.6%	4.4%
SES	Poor	31.3%	21.1%	26.6%
	Average	61.5%	73.0%	66.7%
	Rich	7.3%	5.9%	6.7%
Insurance participation	No insurance	25.3%	15.2%	20.7%
	BPJS	65.6%	82.0%	73.1%
	Private insurance	1.8%	1.2%	1.6%
	Other	7.3%	1.6%	4.7%
Experienced Illness in the Past 12 months	Gout	8.1%	0.0%	4.4%
	Rhematoid Arthritis	18.5%	0.0%	10.1%
	Diabetes	15.1%	14.6%	14.9%
	Hypertension	18.5%	62.4%	38.5%
	CVD	2.3%	2.8%	2.5%
	Common cold	20.1%	8.1%	14.6%
	Respiratory Disease	8.1%	0.0%	4.4%
	Tuberculosis	3.4%	2.5%	3.0%
	Dengue fever	6.0%	2.2%	4.2%
	Other	0.0%	7.5%	3.4%
Illness duration	< 1 week	24.5%	11.5%	18.6%
	2–4 weeks	14.6%	12.1%	13.5%
	> 4 weeks	60.9%	76.4%	68.0%
First treatment	Traditional Medicine	62.2%	10.9%	38.8%
	Transitional Medicine	16.4%	20.5%	18.3%
	Modern Medicine	21.4%	68.6%	42.9%
Nearest healthcare Facility	Traditional healer	52.6%	0.0%	28.6%
	Pharmacy/Drug store	0.8%	18.9%	9.1%
	Private health clinic	15.6%	23.0%	19.0%
	Primary healthcare facility	31.0%	58.1%	43.3%
Total		100.0%	100.0%	100.0%

Majority of the respondents (*N* = 706) belong to the age category 66–79 years old (43.8%), native inhabitants (72.2%), and married (75.6%). Focusing on the difference between urban and rural villages, the gender distribution in the rural (males = 45.1%, *N* = 384) and urban (males = 41.6%, *N* = 322) area is almost equal. Moreover it is clear that the majority of the people in rural villages did not complete any formal education (54.7%) compare to an urban area (30.1%). Regarding the patterns of migration and settlement in the community, the majority of inhabitants in the rural area (89.1%) are native inhabitants. In general, most of the household members were born in the village where they are currently living. However, in an urban area, 38.2% of the respondents were born in other districts (migrants). Katapang, Soreang, and Baleendah as representatives of urban villages in the present study are experiencing urbanisation as are most other urban villages in Kabupaten Bandung. This finding is also supported by the residential status reported by the community members which shows that there are more migrants in urban area (38.2%) than in rural area (14.1%).

In view of the socio-economic profile of the research villages, the economic standard is generally based on the income of the household head or combined with his spouse. Family members also share the responsibility for their family income. The vast majority of respondents in rural and urban area belong to the ‘average’ category 61.5% and 73.0% respectively. The distribution of occupation shows that the majority of the respondents in the rural area are working as farmers (45.8%). However, both of rural and urban area show relatively high on the number of un-employed respondents, 32.6% and 53.7% respectively.

Furthermore, in the rural area, people are mainly treated by traditional healers or used traditional medicine (62.2%) compare to its counterparts, OTC medicine (16.4%) and prescription medicine (21.4%). Conversely, prescription medicine (68.6%) shows as a predominant first treatment choice in the urban area, as opposed to OTC medicine (20.5%) and traditional medicine (10.9%). This phenomenon is also supported by the proximity of health service in the research area. Majority of the respondents in rural area reported ‘traditional healer’ as the nearest health service (52.6%), whereas in urban area, majority of the respondents reported ‘primary healthcare facility’ (58.1%) with no respondent (0.0%) report any of traditional healer near their residence.

### Knowledge and attitude on traditional medicine

Within the context of medical system, knowledge of traditional medicine is not only an understanding of the practices themselves, but also a tapestry of historical, spiritual, and natural philosophies that have withstood the test of time. Attitudes towards traditional medicine, therefore, are as diverse as the cultures from which they emanate, ranging from devout adherence to skeptical apprehension. These attitudes are invariably intertwined with personal beliefs as well as one’s opinion, which conceptualized as block psychosocial factors in the study. Together, knowledge beliefs, and opinions form and attitude that influences the utilization, endorsement, and integration of traditional medicine into daily life and modern healthcare systems. This multifaceted relationship underscores the importance of examining not only the empirical evidence of traditional practices but also the sociocultural dimensions.

Knowledge of traditional medicine is gradually disappearing despite still being practiced in the communities [26]. In view of knowledge, beliefs, and opinion of traditional medicine in West Java, the inhabitants in Kabupaten Bandung are commonly familiar with the medicinal plants and speak profoundly about their knowledge of traditional medicine [27]. The majority of respondents hold much level of knowledge of traditional medicine (Table 2).

**Table 2:** Knowledge, belief, and opinion on traditional medicine

Variable		Rural *N* = 384		Urban *N* = 322		Total *N* = 706	
		N	%	N	%	N	%
Knowledge	Little knowledge	16	4.2%	58	18.0%	74	10.5%
	Average	60	15.6%	63	19.6%	123	17.4%
	Much knowledge	308	80.2%	201	62.4%	509	72.1%
Source of knowledge	Family	337	87.8%	54	16.8%	391	55.4%
	Media	47	12.2%	227	70.5%	274	38.8%
	Healthcare professional	0	0.0%	41	12.7%	41	5.8%
Belief	Little belief	21	5.5%	35	10.9%	56	7.9%
	Average	40	10.4%	44	13.7%	84	11.9%
	Much belief	323	84.1%	243	75.5%	566	80.2%
Opinion	Negative opinion	0	0.0%	14	4.3%	14	2.0%
	Neutral	22	5.7%	30	9.3%	52	7.4%
	Positive Opinion	362	94.3%	278	86.3%	640	90.7%

Table 2 shows that majority respondents in both rural (80.2%) and urban (62.4%) area have much knowledge of traditional medicine. This finding indicates that the Sundanese community in urban area still continues to pass down knowledge of traditional medicine. However, it is also noteworthy that 18.0% of respondents in urban area have little knowledge of traditional medicine and the percentage is higher compared to the respondents in rural area (4.2%). Furthermore, this study finds that majority of the respondents in rural area reported that the source of knowledge of traditional medicine is family members (87.8%). Knowledge of traditional medicine is generally transferred orally, mainly from the parents to their children. However, in urban area majority of respondents reported obtaining the information on traditional medicine from media (70.5%).

Cultural norms and beliefs in natural healing processes are ascribed to the widespread use of traditional medicine [28]. Evidence for the association of spiritual and cultural beliefs with the use of traditional medicine has been shown in the study conducted in Jamaica [29] and Suriname [30]. Likewise, as traditional medicines are deeply rooted in cultural preferences, beliefs in traditional medicine become one of the reasons to use traditional medicine among the Sundanese community. Table 2 indicates that the majority of the respondents rural (84.1%) and urban (75.5%) hold much belief in the efficacy of traditional medicine. Overall, only a minor percentage expresses little belief in traditional medicine (7.9%). Moreover, this study finds that overall respondents commonly show positive opinions on traditional medicine (90.7%). Positive opinions on traditional medicine are associated with being close to home, easy access, affordable, and lower side effects. Many respondents use traditional medicine because it is easy to obtain and cost effective. Furthermore, Gyasi et al. (2016) suggest that respondents’ positive attitudes are associated with the belief in natural remedies [28].

### Utilization of traditional medicine within the plural medical system

The results of the bivariate analysis of are presented in Table 3, arranged in accordance with the different blocks of variables in the conceptual model. The tables show the relationship of each independent variable with the dependent variables.

**Table 3:** Results of bivariate analysis of utilization of medical services

Variable		Traditional Medicine		Transitional Medicine		Modern Medicine		Pear-son χ^2^	Cra-mers V
		N	%	N	%	N	%		
Village category	Rural	266	69.3	39	10.2	79	20.6	0.000	0.292
	Urban	153	47.5	15	4.7	154	47.8		
Illness	Gout	19	61.3	9	29.0	3	9.7	0.000	0.402
	Rhematoid Arthritis	53	74.6	0	0.0	18	25.4		
	Diabetes	75	71.4	0	0.0	30	28.6		
	Hypertension	170	62.5	0	0.0	102	37.5		
	CVD	15	83.3	1	5.6	2	11.1		
	Common cold	48	46.6	33	32.0	22	21.4		
	Respiratory Disease	7	22.6	4	12.9	20	64.5		
	Tuberculosis	2	9.5	0	0.0	19	90.5		
	Dengue fever	17	56.7	0	0.0	13	43.3		
	Other	13	54.2	7	29.2	4	16.7		
Illness duration	< 1 week	82	62.6	26	19.8	23	17.6	0.000	0.231
	2–4 weeks	46	48.4	18	18.9	31	32.6		
	> 4 weeks	291	60.6	10	2.1	179	37.3		
Gender	Male	190	61.9	30	9.8	87	28.3	0.025	0.102
	Female	229	57.4	24	6.0	146	36.6		
Age	0–17 years old	40	64.5	7	11.3	15	24.2	0.067	0.091
	18–65 years old	143	61.4	21	9.0	69	29.6		
	66–79 years old	168	54.4	22	7.1	119	38.5		
	> 80	68	66.7	4	3.9	30	29.4		
Place of birth	In this district	339	60.0	45	8.0	181	32.0	0.503	0.044
	In another district	80	56.7	9	6.4	52	36.9		
Education	No formal education	193	62.9	18	5.9	96	31.3	0.600	0.057
	Basic	149	56.7	22	8.4	92	35.0		
	Intermediate	52	56.5	10	10.9	30	32.6		
	Higher education	25	56.8	4	9.1	15	34.1		
Marital status	Single	48	59.3	7	8.6	26	32.1	0.990	0.014
	Married	317	59.4	41	7.7	176	33.0		
	Divorced/Widowed	54	59.3	6	6.6	31	34.1		
Occupation	Farmer	106	60.2	28	15.9	42	23.9	0.000	0.198
	Construction worker	18	56.3	1	3.1	13	40.6		
	Retailer	53	70.7	7	9.3	15	20.0		
	Private Employee	21	42.9	0	0.0	28	57.1		
	Civil servant	23	51.1	2	4.4	20	44.4		
	Unemployed	184	61.7	16	5.4	98	32.9		
	Other	14	45.2	0	0.0	17	54.8		
Knowledge of Traditional Medicine	Little knowledge	33	44.6	5	6.8	36	48.6	0.050	0.082
	Average	77	62.6	10	8.1	36	29.3		
	Much knowledge	309	60.7	39	7.7	161	31.6		
Source of knowledge I	Family	271	65.5	35	8.5	108	26.1	0.146	0.069
	Media	148	50.7	19	6.5	125	42.8		
	Healthcare personnel	0	0.0	0	0.0	0	0.0		
Belief in Traditional Medicine	Little belief	20	35.7	1	1.8	35	62.5	0.000	0.138
	Average	46	54.8	6	7.1	32	38.1		
	Much belief	353	62.4	47	8.3	166	29.3		
Knowledge of Transitional Medicine	Little knowledge	81	60.9	8	6.0	44	33.1	0.266	0.061
	Average	148	64.3	16	7.0	66	28.7		
	Much knowledge	190	55.4	30	8.7	123	35.9		
Source of knowledge II	Family	183	61.0	18	6.0	99	33.0	0.146	0.069
	Media	157	57.9	19	7.0	95	35.1		
	Healthcare personnel	79	58.5	17	12.6	39	28.9		
Opinion on Transitional Medicine	Negative opinion	17	68.0	3	12.0	5	20.0	0.328	0.057
	Neutral	42	55.3	9	11.8	25	32.9		
	Positive opinion	360	59.5	42	6.9	203	33.6		
Knowledge of Modern Medicine	Little knowledge	208	66.9	29	9.3	74	23.8	0.000	0.171
	Average	112	65.1	11	6.4	49	28.5		
	Much knowledge	99	44.4	14	6.3	110	49.3		
Opinion on Modern Medicine	Negative opinion	21	67.7	5	16.1	5	16.1	0.025	0.089
	Neutral	92	66.2	11	7.9	36	25.9		
	Positive opinion	306	57.1	38	7.1	192	35.8		
Income	0–1.000.000	42	56.8	5	6.8	27	36.5	0.009	0.120
	1.000.001–3.000.000	311	63.1	41	8.3	141	28.6		
	3.000.001-6.000.000	45	46.4	7	7.2	45	46.4		
	6.000.001–9.000.000	12	60.0	1	5.0	7	35.0		
	> 9.000.000	9	40.9	0	0.0	13	59.1		
Expense on medical services	0-500.000	117	54.4	14	6.5	84	39.1	0.030	0.110
	500.001-1.000.000	244	59.4	33	8.0	134	32.6		
	1.000.001-2.000.000	29	78.4	4	10.8	4	10.8		
	2.000.001-3.000.000	23	76.7	2	6.7	5	16.7		
	> 3.000.000	6	46.2	1	7.7	6	46.2		
Insurance participation	No insurance	94	64.4	11	7.5	41	28.1	0.332	0.070
	BPJS	295	57.2	43	8.3	178	34.5		
	Private insurance	7	63.6	0	0.0	4	36.4		
	Other	23	69.7	0	0.0	10	30.3		
Socio-economic status	Poor	124	66.0	11	5.9	53	28.2	0.066	0.079
	Average	263	55.8	42	8.9	166	35.2		
	Rich	32	68.1	1	2.1	14	29.8		
Accessibility on traditional medicine	Easy	364	61.0	47	7.9	186	31.2	0.000	0.122
	Moderate	40	65.6	4	6.6	17	27.9		
	Difficult	15	31.3	3	6.3	30	62.5		
Accessibility on transitional medicine	Easy	204	53.7	21	5.5	155	40.8	0.000	0.136
	Moderate	96	63.2	18	11.8	38	25.0		
	Difficult	119	68.4	15	8.6	40	23.0		
Accessibility on modern medicine	Easy	110	44.5	11	4.5	126	51.0	0.000	0.214
	Moderate	65	56.5	11	9.6	39	33.9		
	Difficult	244	70.9	32	9.3	68	19.8		
Cost to obtain traditional medicine	Cheap	355	60.1	41	6.9	195	33.0	0.357	0.056
	Moderate	20	52.6	3	7.9	15	39.5		
	Expensive	44	57.1	10	13.0	23	29.9		
Cost to obtain transitional medicine	Cheap	252	56.4	30	6.7	165	36.9	0.049	0.082
	Moderate	86	62.3	14	10.1	38	27.5		
	Expensive	81	66.9	10	8.3	30	24.8		
Cost to obtain modern medicine	Cheap	133	51.8	13	5.1	111	43.2	0.000	0.134
	Moderate	96	57.5	15	9.0	56	33.5		
	Expensive	190	67.4	26	9.2	66	23.4		
Residential status	Native	316	62.0	45	8.8	149	29.2	0.009	0.098
	Migrant	93	52.5	8	4.5	76	42.9		
	Temporary	10	52.6	1	5.3	8	42.1		
Nearest health service facility	Traditional healer	142	70.3	23	11.4	37	18.3	0.000	0.152
	Pharmacy/Drug store	34	53.1	5	7.8	25	39.1		
	Private health clinic	80	59.7	4	3.0	50	37.3		
	Primary healthcare	163	53.3	22	7.2	121	39.5		
Farthest health service facility	Traditional healer	45	42.9	3	2.9	57	54.3	0.000	0.166
	Pharmacy/drug store	2	28.6	0	0.0	5	71.4		
	Private health clinic	2	66.7	1	33.3	0	0.0		
	Primary healthcare	86	69.4	11	8.9	27	21.8		
	Hospital	284	60.8	39	8.4	144	30.8		
Impact of BPJS	No Impact	85	72.6	10	8.5	22	18.8	0.000	0.141
	Less Impact	85	69.1	12	9.8	26	21.1		
	Much Impact	249	53.4	32	6.9	185	39.7		
Impact of promotion on traditional medicine	No impact	347	62.3	38	6.8	172	30.9	0.007	0.118
	Less impact	72	48.3	16	10.7	61	40.9		
	Much Impact	0	0.0	0	0.0	0	0.0		
Impact of promotion on transitional medicine	No impact	350	64.2	42	7.7	153	28.1	0.000	0.165
	Less impact	54	42.9	5	4.0	67	53.2		
	Much impact	15	42.9	7	20.0	13	37.1		
Total		419	59.3	54	7.7	233	33.0		

Within the category of psycho-social variables, the variable ‘belief in traditional medical system’ reveals a strongly significant relationship (χ^2^ = 0.000), with Cramer’s V (V = 0.138) which indicates the association between two variables. In general, patients who admitted to having much belief (62.4%, *n* = 353) in traditional medicine have more frequent contact with the traditional medical system than other medical services, in contrast to patients with little belief (62.5%, *n* = 35) who seek treatment from the modern medical system more frequently than average others.

Furthermore, ‘impact of public health insurance (BPJS)’ (χ^2^ = 0.000, V = 0.141) and ‘impact of the promotion on transitional medicine’ (χ^2^ = 0.000, V = 0.165) demonstrate the most strongly significant relationship over the dependent variables with Cramer’s V value indicating acceptable association within the category of intervening variables. In general, patients who admit that public health insurance (BPJS) has no impact (72.6%, *n* = 85) on transcultural health care utilization use traditional medicine more frequently than average (39.4%, *n* = 241). In addition to the variables of BPJS participation, the variable ‘promotion on the transitional medicine’ shows a very strongly significant relationship (χ^2^ = 0.000, V = 0.165) over the dependent variable with Cramer’s V value indicating the minimally acceptable association.

## Discussion

Health care utilization behaviour involves the decision-making process at the community or household level. Understanding the pattern of people’s health care utilization behaviour helps to improve health outcomes within the population. In addition, information on health-seeking behaviour and patterns of health care utilization will provide assistance in health care policy planning prevention and management of health conditions [21, 22]. As an archipelago inhabited by great numbers of ethnic groups, Indonesia is characterized by the plural medical system to suit the varied needs of the people. With regards to utilization of medical systems, the traditional medicine presents as the dominant medical system in the research area, accounting for 59.3% (*n* = 419) of total utilization, followed by the modern medicine (33.0%, *n* = 233), and transitional medicine (7.6%, *n* = 54). Medical pluralism is not a new concept for regions where there is a diffusion of cultural and social medical systems, such as in the Asian region. A study of the National Health Survey in Taiwan reveals that 32.5% of the population has reported the use of multiple healing systems including modern Western medicine, Traditional Chinese Medicine, as well as religious or spiritual healing [7].

The results have established that factors such as village category, health condition (type of disesase), duration of the disease, and occupation are significant factors (χ^2^ = 0.000) in health care utilization among community members. While variables such as gender, age, and education are not identified as significant factors in the present study. However, several studies have reported some inconsistent findings regarding those variables. In a review on the utilization of traditional, complementary, and modern medicine in Indonesia, Pengpid & Peltzer (2019) conclude that several sociodemographic and health-related factors such as age (older), religion (*Muslim*), environment (urban area), health condition (unhealthy), and having chronic conditions were associated with the use of traditional, complementary and/or modern medicine [31]. Although gender has not been identified as a significant determinant in health care utilization in the present study, several studies in different community settings report otherwise. A recent study in Malaysia reveals that women have higher utilization rates of primary care than men [32]. Another noteworthy finding is that while gender is not a significant determinant variable in this study, among household members, health care utilization is generally decided by women as a mother or wife instead of men as the household head.

According to the findings, different behaviours are considered by patients in order to diagnose, control and improve their own disease. The socio-demographic characteristics of the respondent relate to levels of utilization. Educational status helps determine whether a decision to choose one of the medical systems is influenced by this variable. Several studies indicate that education has an important impact on health care utilization [33]. Although there is no considerable difference among categories within the variable ‘education’ in the utilization of the traditional medical system, the cross-tabulation table reveals that patients without formal education (62.9%, *n* = 193) used traditional medicine more frequently than average. Many of those who have higher-level education beliefs and have a positive opinion of traditional medicines often prefer modern medicine due to scientific efficacy and safety. A study conducted by Rasul et al. (2019) on determinants of health-seeking behaviour for non-communicable disease in Bangladesh reveals that higher education, major chronic non-communicable disease, higher socio-economic status, lower proportion of chronic household patients, and shorter distance between a household and a sub-district public referral health facility increased the likelihood of seeking a modern health care provider than its counterpart [34]. Furthermore, the diverse patterns of health care utilization among community members are found across the different levels of education. In general, people with basic education generally use transitional medicine. Although there is no considerable difference among categories within the utilization of the traditional medical system, people with a higher level of education tend to use traditional medicine more frequently than average. This finding is in contrast with a study conducted by Peltzer & Pengpid (2019) which found the association of lower education with the utilization of traditional medicine [31].

In a study on perceptions of the effectiveness of traditional medicine in Ghana, Gyasi et al. (2011) report that traditional medicine is perceived to be effective by patients for the treatment of broken bones, impotence, infertility, mental disorder and hypertension, while a lack of belief and negative opinions in the effectivity of traditional medicine is associated with insufficient scientific data regarding its safety [28]. These findings are in agreement with the study conducted in the Kilimanjaro region, where some of the respondents perceived traditional medicine to be unsafe and could damage organs [12]. In the research area, patients generally contact modern medical services in severe/emergency conditions.

Most studies found an association of residential characteristics with the use of traditional medicine [30, 34]. This study also finds that characteristics of external environment and community such as rural/urban community, residential status, and geographical conditions significantly influence utilization of medical systems; being native and residing in rural areas and highlands are associated with more frequent contact with traditional rather than transitional and modern medicine. However, in their comparative study, Oyebode et al. (2016) report mixed results regarding the influence of external environment factors on the utilization of traditional medicine. While in China rurality is associated with the use of traditional medicine, Ghana and India show the opposite results [35]. A study conducted by Nurhayati & Widowati (2017) also reveals that households who reside in urban areas were more likely to use traditional medicine [36].

The rather easy access to traditional medicine in the village is one of the reasons it is still being used. In the past, the hospital was too far from the village. Back then only traditional medicine was used. Nowadays the roads are more accessible and thus the hospital is more easily reached. Besides that, small hospitals are being built in some villages, so even near some of the smallest villages, there is a hospital.

Furthermore, this study reveals that regardless of the proximity to health providers, people are open to whichever medical treatment they consider the best. Patients are flexible to combine different but complementary treatment to achieve better results. Similarly, in a study related to distance and health care utilization, Mattson (2010) reveals that for chronic conditions, distance and transportation did not significantly influence number of visits to formal health care facilities.

Policy on medical systems, public insurance, and promotion on transitional medicine play key roles since policymakers are often interested in understanding the influence of health policy on utilization of medical systems. Extensive studies on health care policies have shown that utilization patterns vary across uninsured and insured community members. Comparatively, the study in Ghana also reports that households in both rural and urban areas who have public health insurance prefer modern medicine over traditional medicine [35].

This study revealed that most of the knowledge of herbal remedies is handed down by elders in the community to the younger members. This finding demonstrates that ethnomedicinal knowledge is concentrated in the elder community members and its transfer to the younger generation is relatively difficult and slow. This phenomenon might be affected by modernization and environmental change. Similar results were shown in a study in Ethiopia where the elderly group demonstrated higher knowledge of medicinal plants than youngsters. Modern education is assumed to make the younger generation underestimate local knowledge. Thus, age and education appear to be the main factors in the level of traditional knowledge [26, 37, 38]. Furthermore, migration of the young generation to the cities also presents a challenge in the continuation of traditional medical knowledge, as the urban community tends to undermine cultural beliefs and traditional knowledge [26]. Likewise, a study in Morocco also reveals that knowledge of medicinal plants is in danger because of the influence of modernization, resulting in the mistrust of young generations to trust traditional medicine [39].

Given together, this study highlights the importance to study of local people knowledge, belief, and practice of the traditional medical system within plural medical system in the community. Determinants identified in the study will fill a gap in public health knowledge which contributes to improvements in health planning. Moreover, the study on the local concepts of health and illness helps improve health outcomes in the research area because health care practitioners and policy makers may have a better understanding of the health beliefs of local people, and potentially integrate local concepts of health and illness into their work.

The data of the study will be useful in planning and developing effective public health intervention resulting in decreased physical and social burden for the target population. In the present study, determinants which influence health care utilization will help in identifying barriers to the successful implementation of public health intervention in the target population.

The community-based approach in the transcultural health care utilization of the present study elucidates the *emic* perspective of health care needs and their limitations. The results are more generalizable across regional populations. With this regard, the analysis of the results of this study has a predictive value for future health care planning. Furthermore, this study presents each medical system which co-exists in the research area within the socio-cultural context, therefore generating a practical community-oriented perspective on medical pluralism.

### Limitation and strength

The self-reported nature in this subset of data might pose recall and social-desirability biases. Moreover, variables such as beliefs and opinions are deeply rooted in the specific cultural context of the study area. This could limit the generalizability of the findings to other settings. This study also excludes the staging of disease severity.

The strength of this study lies in its comprehensive approach to understanding healthcare utilization behavior within a plural medical system, particularly focusing on the community or household decision-making process.

## Conclusion

In a society as diverse as Indonesia, traditional medicine is still widely used for the treatment of various chronic diseases. In general, the population in Kabupaten Bandung seeks treatment from various sources, which in the context of the medical system, consists of the traditional, transitional, and modern medical system; therefore, it adopts the patterns of transcultural health care utilization. In terms of the knowledge, beliefs, and practices of traditional medicine in West Java, the inhabitants of the five research villages were commonly familiar with medicinal plants and speak profoundly about their knowledge of traditional medicine, which in the research area is perceived as accessible, efficacious, affordable and culturally appropriate with Sundanese community. With regards to the total utilization by patients, the traditional medical system presents as the dominant medical system in the research area. The results of the bivariate analysis indicate that out of thirty eight identified variables within the multivariate model, fourteen variables show a significant relationship with the health care utilization variable namely: village category, illness, illness duration, occupation, belief in traditional medicine, knowledge of modern medicine, accessibility, cost, proximity to the medical service, and insurance.

## Data Availability

The datasets generated and/or analysed during the current study are available in the *Data Archiving and Networked Services (DANS)* repository with persistent identifier: 10.17026/dans-xdq-xfp8. (available online at: https://ssh.datastations.nl/dataset.xhtml?persistentId=doi).
